# Breast Lesion Classification with Multiparametric Breast MRI Using Radiomics and Machine Learning: A Comparison with Radiologists’ Performance

**DOI:** 10.3390/cancers14071743

**Published:** 2022-03-29

**Authors:** Isaac Daimiel Naranjo, Peter Gibbs, Jeffrey S. Reiner, Roberto Lo Gullo, Sunitha B. Thakur, Maxine S. Jochelson, Nikita Thakur, Pascal A. T. Baltzer, Thomas H. Helbich, Katja Pinker

**Affiliations:** 1 Memorial Sloan Kettering Cancer Center, Department of Radiology, Breast Imaging Service, New York, NY 10065, USA; reinerj@mskcc.org (J.S.R.); logullor@mskcc.org (R.L.G.); thakurs@mskcc.org (S.B.T.); jochelsm@mskcc.org (M.S.J.); pinkerdk@mskcc.org (K.P.); 2 Department of Radiology, Breast Imaging Service, Guy’s and St. Thomas’ NHS Trust, Great Maze Pond, London SE1 9RT, UK; 3 Memorial Sloan Kettering Cancer Center, Department of Medical Physics, New York, NY 10065, USA; 4 Touro College of Osteopathic Medicine, Middletown, NY 10940, USA; nthakur@student.touro.edu; 5 Department of Biomedical Imaging and Image-Guided Therapy, Division of Molecular and Structural Preclinical Imaging, Medical University of Vienna, 1090 Wien, Austria; pascal.baltzer@meduniwien.ac.at (P.A.T.B.); thomas.helbich@meduniwien.ac.at (T.H.H.)

**Keywords:** magnetic resonance imaging, breast neoplasms, machine learning, diffusion magnetic resonance imaging, multiparametric magnetic resonance imaging

## Abstract

**Simple Summary:**

Currently, breast contrast-enhanced MRI is the most sensitive imaging technique for breast cancer detection; however, its specificity is low given the common characteristics shared by benign breast lesions and some cancers. This leads to a high number of false-positive cases and, therefore, unnecessary biopsies. Multiparametric MRI including diffusion-weighted imaging assists in this task by increasing the specificity for breast lesion discrimination. Nevertheless, interpretation of breast MRI is still highly dependent on the reader’s level of experience. Our work combines radiomic features extracted from multiparametric MRI to generate predictive models for breast cancer differentiation. Additionally, decision support models were compared with the performance of two breast dedicated radiologists for lesion differentiation. Our work proves the potential of multiparametric radiomics coupled with machine learning to be implemented in clinical practice for lesion differentiation on breast MRI. AI algorithms show value to assist less experienced readers, improving the accuracy for breast lesion discrimination.

**Abstract:**

This multicenter retrospective study compared the performance of radiomics analysis coupled with machine learning (ML) with that of radiologists for the classification of breast tumors. A total of 93 consecutive women (mean age: 49 ± 12 years) with 104 histopathologically verified enhancing lesions (mean size: 22.8 ± 15.1 mm), classified as suspicious on multiparametric breast MRIs were included. Two experienced breast radiologists assessed all of the lesions, assigning a Breast Imaging Reporting and Database System (BI-RADS) suspicion category, providing a diffusion-weighted imaging (DWI) score based on lesion signal intensity, and determining the apparent diffusion coefficient (ADC). Ten predictive models for breast lesion discrimination were generated using radiomic features extracted from the multiparametric MRI. The area under the receiver operating curve (AUC) and the accuracy were compared using McNemar’s test. Multiparametric radiomics with DWI score and BI-RADS (accuracy = 88.5%; AUC = 0.93) and multiparametric radiomics with ADC values and BI-RADS (accuracy= 88.5%; AUC = 0.96) models showed significant improvements in diagnostic accuracy compared to the multiparametric radiomics (DWI + DCE data) model (*p* = 0.01 and *p* = 0.02, respectively), but performed similarly compared to the multiparametric assessment by radiologists (accuracy = 85.6%; AUC = 0.03; *p* = 0.39). In conclusion, radiomics analysis coupled with the ML of multiparametric MRI could assist in breast lesion discrimination, especially for less experienced readers of breast MRIs.

## 1. Introduction

Medical imaging has always played a pivotal role in breast cancer diagnosis and treatment decision-making. The inherently high sensitivity of dynamic contrast-enhanced magnetic resonance imaging (DCE-MRI) (81–100%) [1] had led to its wide use in the evaluation of breast cancer, with many indications. Despite its powerful ability to identify abnormalities in the breast, DCE-MRI has limitations, such as reduced availability, high cost, and a reduced pooled specificity of 70% [2,3,4,5,6,7].

Multiparametric MRI schemes that integrate diffusion-weighted imaging (DWI) achieve better specificity than DCE-MRI alone, reducing the number of false-positive biopsies [8,9,10,11,12,13,14,15,16]. Nevertheless, the clinical standardization of DWI as a supportive MRI sequence remains challenging, given the broad inconsistency of protocols and interpretative methods, as well as interobserver variability [17,18].

In the last decade, radiomics has become an area of increasing interest. As a technique that mines quantitative imaging features that are hidden to the radiologist’s eye, radiomics can be combined with clinical data (e.g., histopathologic, genomic, or molecular information) and artificial intelligence (AI) to build algorithms that are capable of emulating the human brain in tasks of learning and problem solving. To date, the development of such decision-support algorithms for breast cancer evaluation has mainly relied on radiomics data derived from DCE-MRI. This development has had applications for the characterization of different molecular profiles of breast cancer [19,20], the prediction of likelihood for axillary lymph node metastatic involvement [21], and the probability of tumor response to chemotherapy treatment [22], as well as the differentiation between breast lesions [23,24,25,26,27,28,29]. However, the facets of clinical implementation of these support decision models are still to be determined, particularly in the setting of multiparametric MRI.

The purpose of this multicenter retrospective study was to evaluate the diagnostic value of radiomics coupled with machine learning (ML) of multiparametric MRI as used in the clinical routine by comparing its performance with that of experienced radiologists in the classification of enhancing breast tumors. Multiparametric MRI-based algorithms could help less experienced breast MRI readers in the task of breast lesion differentiation.

## 2. Materials and Methods

### 2.1. Study Sample

This institutional review board-approved multicenter retrospective study was conducted in compliance with the United States Health Insurance Portability and Accountability Act. The need for written informed consent was waived. Some patients were previously reported on in a different context [8,30].

**Figure 1:**
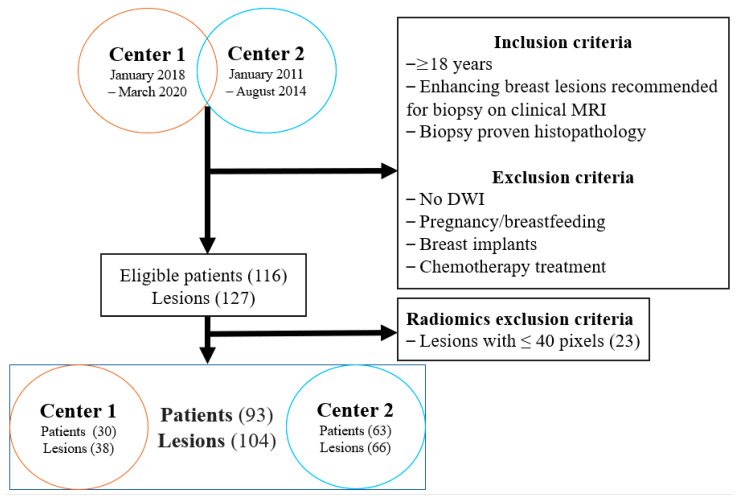
Flowchart for the selection of patients in the study. DWI, diffusion-weighted imaging.

Consecutive patients were identified following a review of databases from Memorial Sloan Kettering Cancer Center (MSK) spanning the period from January 2018–March 2020, and the Medical University of Vienna (MUV) spanning the period from January 2011–August 2014. Figure 1 illustrates the selection of the patients included in the study. Inclusion and exclusion criteria are described in the Supplementary Materials.

### 2.2. Breast MRI Technique

At MSK, 3 T MRI scanners (GE Discovery 750, GE Healthcare, Chicago, IL, USA) using 8-channel (13/93 examinations, 14%) or 16-channel breast coils (Sentinelle coils, Hologic, Marlborough, MA, USA) (20/93 examinations, 21%) were used. The breast MRI protocol comprised fat-suppressed T2-weighted fast spin echo imaging and fat-suppressed 3D T1-weighted imaging using differential subsampling with Cartesian ordering (DISCO) before and after contrast agent injection (0.1 mmol gadobutrol/kg body weight). DWI was performed using two different sequences: single-shot echo-planar with parallel imaging array spatial sensitivity encoding technique (ASSET) (22/33 examinations, 66.7%) and multishot multiplexed sensitivity-encoding (MSUE) (11/33 examinations, 33.3%). At MUV, the scans were performed on a 3 T MRI scanner (Tim Trio, Siemens, Erlangen, Germany) using 4-channel breast coils (InVivo, Orlando, FL, USA) (60/93 examinations, 65%). The protocol was similar including fat-suppressed T2-weighted turbo spin echo imaging, fat-suppressed DCE T1-weighted imaging before and after contrast injection (0.1 mmol gadoterate meglumine/kg body weight). DW images were acquired using a readout-segmented echo planar encoding scheme.

DWI was acquired consistently before gadolinium-based contrast injection and the apparent diffusion coefficient (ADC) maps were obtained using a built-in software. The structure and parameters for both MRI protocols are presented in the Supplementary Materials (Tables S1 and S2).

### 2.3. Imaging Evaluation and Processing

Lesions were manually segmented for radiomics analysis. Two breast radiologists (IDN and RLG), each one with five years level of experience in breast imaging, reviewed Digital Imaging and Communications in Medicine (DICOM) images from early post contrast-enhanced T1-weighted imaging, DWI, and ADC mapping in consensus to segment lesions. Lesions from the three sets of images were matched on OsiriX viewer v 9.0, and the slice containing the largest lesion diameter was recorded. Subsequently, one 3D segmentation was performed on each set of images by using the online ITK-SNAP v 3.6.0 tool. The same number of segmentations was performed per radiologist by delineating the borders of each lesion in every slice where it was visible to obtain a volume of interest (VOI). In the case of DW images, VOIs were directly extrapolated to ADC maps and manually corrected in the case of mismatched areas for feature extraction.

**Figure 2:**
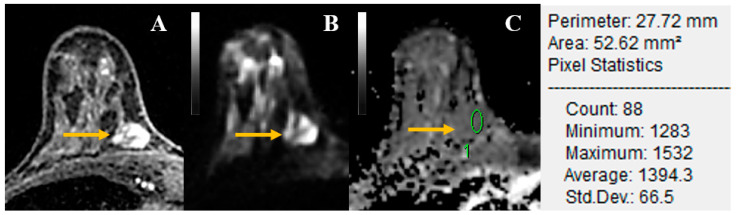
Axial MR images of a 48-year-old woman with a 14-mm benign mass in the right breast, of which biopsy yielded fibro-adenomatoid changes (yellow arrows). (**A**) Axial dynamic contrast-enhanced image depicts a heterogeneous, oval, and circumscribed enhancing mass in the right breast corresponding to a heterogeneous hyperintense lesion on axial diffusion-weighted imaging (DWI) at a b value of 800 s/mm^2^; (**B**). (**C**) Correlative parametric apparent diffusion coefficient (ADC) map with a region of interest (ROI) placed within the darkest part of the lesion and ROI information. ADC values are expressed in mm^2^/s. This lesion was heterogeneous vs. non-enhancing septa and therefore characterized as BI-RADS 3 and 3 based on the DWI score in the consensus reading of radiologists.

One month after segmentation, independent reads of the multiparametric MRI images were performed. Two radiologists (IDN and JSR) with five and six years of experience in breast imaging, respectively, rated cases in two sessions separated by at least three weeks. The radiologists were blinded to the patients’ selection criteria, histopathological results, and previous imaging. In the first reading session, DW images and corresponding ADC maps were reviewed by each radiologist, using the previously recorded slice containing the largest lesion diameter as a reference. A category for suspicion (1—very low, 2—low, 3—intermediate, 4—high, 5—very high) was assigned according to the signal intensity of the lesions on DW images (b = 800 s/mm^2^). Lesions assigned a category ≥4 were considered positive for malignancy. Additionally, the corresponding ADC values on ADC maps (for ADC values, a cut-off of 1.3 × 10^−3^ mm^2^/s were noted. Lesions with ADC values above the cut-off were considered positive for malignancy, as recommended by the European Society of Breast Imaging international breast diffusion-weighted imaging Working Group [17]. An example of region of interest placement to obtain ADC values is shown in Figure 2.

In the second reading session, DCE images were assessed using BI-RADS. Like the suspicion score for the DW images, lesions assigned a category for suspicion ≥ 4 based on BI-RADS were considered positive for malignancy. Additionally, a multiparametric MRI classification combining BI-RADS categories and ADC values was performed. In cases of discrepancy between the suspicion level for BI-RADS categories and DWI scores, ADC with a cut-off 1.3 × 10^−3^ mm^2^/s was used to classify the lesions.

In a third reading session, consensus analysis of the two radiologists for all cases regarding the level of suspicion was made for the BI-RADS, DWI score, and multiparametric MRI suspicion score. This allowed for comparison of the radiologists’ performance to that of the ML models. ADC values obtained by the radiologist with more experience in using DWI (IDN) were used for consensus on the multiparametric MRI suspicion score. Additionally, BI-RADS descriptors for enhancing lesions were noted for mass and non-mass enhancement (NME) lesions as shown in Table 1.

### 2.4. Radiomics Analysis

The information extracted from the VOIs derived from DCE and DW images was entered into the Computational Environment for Radiological Research (CERR) software (available on Github) using an in-house MATLAB (MathWorks Inc., Natic, MA, USA) code. CERR then allowed for the calculation of radiomic features [31], based on the grey level run length matrix (RLM), grey level co-occurrence matrix (GLCM), grey level size zone matrix (SZM), neighborhood grey tone difference matrix, neighborhood grey level dependence matrix, and first-order statistics.

Data reduction to 16 grey levels was performed to account for the reduced number of pixels in some lesions. To ensure sufficient counting statistics for the calculation of texture features, only a distance of one was regarded between pixels. To optimize the models, lesions containing less than 40 pixels were disregarded. As a result, 23 patients with 23 lesions were excluded. The final study sample consisted of 93 patients (30 from MSK and 63 from MUC) with 104 lesions (38 from MSK and 66 from MUV), with 11 patients showing more than one lesion on MRI.

### 2.5. Reference Standard

The reference standard was histopathology obtained through image-guided biopsy in all lesions, whether MRI (30 lesions) or ultrasound-guided (74 lesions). In cases of histopathology that yielded a benign but high-risk lesion (e.g., atypical ductal or lobular hyperplasia or papilloma), the postsurgical histopathology report was consulted to verify concordance with results from image-guided biopsy.

### 2.6. Statistical Analysis and Predictive Model Building

Means (±SD) and medians (range) were used to define continuous variables whereas proportions were used to summarize categorical variables.

**Figure 3:**
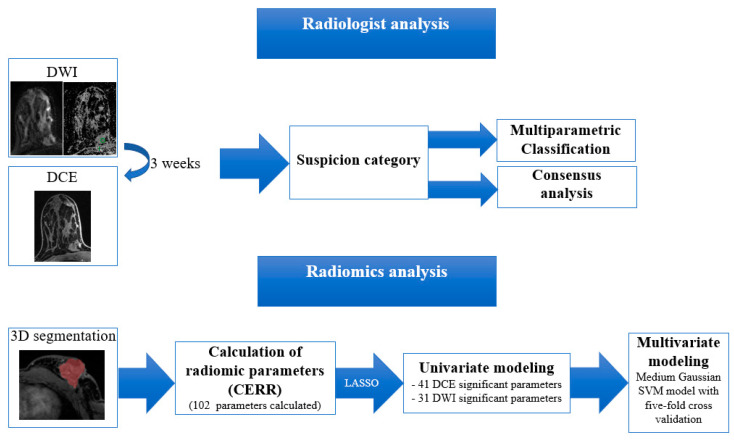
Workflow for the radiomics analysis. DCE-MRI, dynamic contrast-enhanced MRI; DWI, diffusion-weighted imaging; CERR, Computational Environment for Radiological Research; LASSO, least absolute shrinkage and selection operator; SVM, support vector machine.

Prior to statistical analysis (SPSS version 25, IBM Corp., Armonk, NY, USA), ComBat harmonization was performed to reduce possible variability between the different MRI protocols used [32]. Afterwards, statistical univariable modelling afforded the identification of significantly different radiomic features between benign lesions and cancers. To prevent model overfitting, feature selection was performed using a fivefold cross-validated elastic net and combining least absolute shrinkage and selection operator (LASSO) regression and ridge regression. We selected the top five radiomic features to ensure sufficient cases per feature for model building of the minority class. Multivariate modeling through medium Gaussian support vector machine (SVM) modelling with fivefold cross-validation afforded the generation of robust ML models for breast lesion differentiation. Z-score normalization of the radiomic parameters was used for model building in consideration of the different degrees of magnitude found in radiomics. Figure 3 shows the workflow for radiomics and radiologist analysis.

The area under the receiver operating characteristic curve (AUC) and accuracy were used to assess the models’ performance. Diagnostic accuracies were compared using McNemar’s test, and *p*-values < 0.05 were considered significant. Sensitivity, specificity, positive predictive value (PPV), and negative predictive value (NPV) were calculated for both radiologists and models. Diagnostic metrics were obtained for mass and NME lesions together, as well as for masses alone, allowing for the evaluation of models that utilize individual BI-RADS descriptors (internal enhancement, shape, margins, enhancing kinetics).

## 3. Results

### 3.1. Patient Sample and Breast Lesion Characteristics

A total of 93 women (mean age: 48.5 years ± 12 years) with 104 lesions (mean size: 22.8 ± 15.1 mm) were included in the final patient sample. There were 46 cancers (mean size: 28.8 ± 18.2 mm), of which 35 were mass lesions and 11 were non-mass enhancements. Benign lesions accounted for 58 lesions (mean size: 18.2 ± 10 mm), of which 50 were mass lesions and 8 were non-mass enhancements. Patient and lesion characteristics are summarized in Table 2 and Table 3.

### 3.2. Radiomics Analysis for Breast Lesion Differentiation

The median size of segmented lesions was 255 pixels (range: 40–5379 pixels) for benign lesions and 2104 pixels (range: 115–58,485 pixels) for malignant lesions.

After CERR analysis, 102 radiomic features were obtained: 22 based on first-order statistics; 26 based on GLCM; 16 based on RLM; 16 based on SZM; 17 based on neighborhood grey level dependence matrix; and 5 based on neighborhood grey tone difference matrix.

Univariable analysis yielded 34 DWI and 27 DCE radiomic features that were significantly different between benign and malignant lesions. Feature selection, followed by multivariable modelling, resulted in ten models for the classification of all lesions, as well as for the classification of mass lesions alone. The top five radiomics parameters selected to develop each model are provided in Tables S3 and S4.

### 3.3. Radiologist Performance vs Radiomics Coupled with ML for Malignant vs. Benign Classification for Mass Lesions

The performance of radiologist consensus reading, as well as that of different models for the classification of mass lesions, are shown in Table 4. The “radiomics DWI data model” demonstrated a higher diagnostic accuracy for the classification of mass lesions based on DWI (78.6%, CI: 68.3–86.8%) than either the ADC value (73.8%, CI: 63.1–82.8%) or the DWI score (75.0%, CI: 64.4–83.8%) assessed by radiologists. However, this increase in diagnostic accuracy was not significant (*p* > 0.38). Both the “radiomics DWI data with DWI score model “(78.6%, CI: 68.3–86.8%) and the “radiomics DWI data with ADC value model” (82.1%, CI: 72.3–89.7%) did not lead to a significant improvement in diagnostic accuracy when compared with the “radiomics DWI data model” (*p* > 0.39 for both).

For the classification of mass lesions based on DCE, the “radiomics model using individual BI-RADS descriptors for masses” demonstrated a significant improvement in diagnostic accuracy (86.9%, CI: 77.8–93.3%) compared with BI-RADS (classic DCE-MRI) scoring as assessed by radiologists (71.4%, CI: 60.5–80.8%; *p* = 0.01), the “radiomics DCE data” model (71.4%, CI: 60.5–80.8%; *p* = 0.013), and the “radiomics DCE data with BI-RADS” model (77.4%, CI: 67.0–85.8%; *p* = 0.007). The “radiomics DCE data with individual BI-RADS descriptors for masses model” offered no further significant improvement in diagnostic accuracy (86.9%, CI: 77.8–93.3%; *p* = 1.00).

For the classification of mass lesions based on the multiparametric assessment of DWI and DCE data, the “multiparametric radiomics with ADC values and individual BI-RADS descriptors for masses model” provided a significant improvement in diagnostic accuracy (91.7%, CI: 83.6–96.6%) compared with the “multiparametric radiomics (DWI and DCE data) model” (79.8%, CI: 69.6–87.8%; *p* = 0.03). However, the “multiparametric radiomics with ADC values and individual BI-RADS descriptors for masses model” was not significantly different compared to multiparametric MRI (ADC value with BI-RADS) assessment by radiologists (86.9%, CI: 77.8–93.3%; *p* = 0.06).

### 3.4. Radiologist Performance vs. Radiomics Coupled with ML for Malignant vs. Benign Classification for All Lesions (Mass and Non-Mass Lesions)

For detailed results regarding radiologist performance vs. radiomics coupled with ML performance for the classification of all lesions together (mass and non-mass enhancement), see the Supplementary Data (Tables S5–S7).

## 4. Discussion

We investigated the diagnostic performance of different models for the classification of enhancing breast tumors that were deemed suspicious on routine clinical breast MRI evaluation and subsequently recommended for biopsy. We compared the performance of radiomics analysis coupled with machine learning models against that of dedicated breast radiologists. A total of ten models were developed that used radiomic features extracted from DCE and ADC maps derived from DW images with clinical information (e.g., BI-RADS category, BI-RADS descriptors, or DWI-derived data) to discriminate between malignant and benign breast lesions.

Our results showed that multiparametric MRI interpretation by radiologists, as well as radiomic models based on multiparametric MRI combined with BI-RADS and DWI clinical data, achieved the highest accuracies and AUC values. While yielding slightly higher diagnostic accuracies, the multiparametric radiomics models with BI-RADS and ADC values did not significantly improve upon the diagnostic accuracy of dedicated study radiologists. It must be noted that all the lesions evaluated in this study had been previously classified as suspicious on routine reads and were already recommended for biopsy, indicating that such AI-enhanced multiparametric MRI models would be promising in clinical practice where readers of all levels of experience are reading breast MRIs.

Regarding non-multiparametric assessments, the models based on DWI features did not improve upon radiologist performance using DWI alone. Based on DCE, only the “radiomics DCE data with BI-RADS model” provided a borderline significant improvement in diagnostic accuracy when compared with radiologists’ assessment of breast lesions using BI-RADS classification. This may be due to the addition of an algorithmic/decision-tree component to the subjective assessment with BI-RADS. This trend was sustained, and a significant improvement was observed when the actual BI-RADS descriptors (internal enhancement, shape, margins, enhancing kinetics) were incorporated into a radiomics model based on DCE in the classification of mass lesions.

Multiparametric breast MRI with DCE-MRI and DWI as a supportive sequence for the discrimination of breast lesions is shown to be the best imaging technique for breast cancer diagnosis [33,34]. Yet only a few studies have been published comparing the performance of AI-enhanced models to that of radiologists for breast cancer diagnosis. This information is key for the implementation of these models in clinical practice. We showed that diagnostic accuracy of radiologists using multiparametric MRI with ADC values (85.6%; *p* = 0.39) can be improved through use of a multiparametric radiomics model with BI-RADS and ADC values (88.5%, CI: 80.7–93.9%). This was further emphasized when we analyzed the subgroup of masses, in which the multiparametric radiomics model with individual BI-RADS descriptors and ADC values provided borderline significance when compared with the accuracy of radiologists based on multiparametric MRI using ADC values (91.7% vs. 86.9%; *p* = 0.063). This result is unsurprising given that non-mass lesions usually present as diffuse infiltrating enhancements with ill-defined margins and often represent a diagnostic challenge for both manual segmentation and DWI scoring [35].

Among the few studies directly comparing AI-enhanced models and radiologists, Sutton et al. [36] proved that quantitative radiomic features extracted from DCE-MRI of breast cancer could replicate human-extracted tumor size and BI-RADS imaging phenotypes. Another study, conducted by Truhn et al. [37], assessed the performance of a convolutional neural network (CNN) model against radiomics analysis, comparing it with the prospective assessment of three breast radiologists discriminating breast MRI enhancing lesions. The CNN model seemed to outperform radiomics analysis (AUC of 0.88 vs. 0.81) but did not achieve better performance than multiparametric MRI interpreted by breast radiologists (AUC of 0.98). Unlike our study, the input to perform radiomics analysis and to generate the CNN model was only from features extracted from DCE images, and thus not representative of the full diagnostic potential of breast multiparametric MRI. Lo Gullo et al. [38] compared the qualitative morphological assessment with BI-RADS classification to radiomics coupled with machine learning for the differentiation of subcentimeter breast masses in BRCA mutation carriers. They found that radiomics analysis coupled with ML achieved a better diagnostic accuracy (81.5%) compared with radiologists using BI-RADS classification (53.4%). In yet another study, Bickelhaupt et al. [27] investigated two radiomics classifiers based on contrast-free MRI sequences (DWI and T2-weighted sequences) alone, and combined with ADC parameter, for the discrimination of breast lesions found suspicious on screening mammography. As in our study, they reported that their radiomics models performed better than ADC alone and that the inclusion of the mean ADC increased the accuracy of the model (from an AUC of 0.842 to 0.851), demonstrating the advantages of data sharing. Nevertheless, the performance of the proposed model combined with ADC was lower than that of expert breast radiologists (AUC of 0.959) using multiparametric breast MRI.

It is worth noting some of our study’s strengths. First, our study included data from 3D segmentations, which contributes more pixels and thereby enables better model building and accuracy. Moreover, our data are derived from images acquired from different scanners and MRI protocols across two different institutions. Although this could be understood as a weakness, e.g., the introduction of data noise or dilution of the association by the protocol/image quality differences, it is helpful for the generalizability of our results. Secondly, our study’s readers were experienced and breast-dedicated radiologists. Their excellence in assessing the lesions certainly impacted on our results. Therefore, our study indicates potential for AI-enhanced multiparametric MRI to be useful in clinical practice as a decision support tool for readers of all levels of experience or as support for breast radiology residents and fellows. Having said that, it is important to highlight that breast MRI has a high cost, and its access may be limited in certain countries. Therefore, it is important to quest for alternative AI-enhanced tools with wider availability. New approaches such as ultrasound elastography coupled with machine learning techniques may represent a feasible alternative to MRI for breast cancer diagnosis [39].

Regarding limitations, our study included a relatively small representative sample comprised of 104 breast lesions. This small sample size precluded the separation of data into training and test sets. In addition, some of these lesions, particularly benign tumors, were subcentimeter, which may affect the number of pixel contributions for feature extraction and lead to an increased proportion of features that were potentially contaminated by partial volume effects. We tried to overcome this limitation while ensuring adequate counting statistics by including only lesions with more than 40 pixels and lowering the data to only 16 grey levels (vs. 32 or 64 grey levels, as previously employed in breast MRI).

## 5. Conclusions

In conclusion, multiparametric radiomics analysis coupled with ML and combined with clinical data from multiparametric MRI performed similarly to breast radiologists for the classification of breast enhancing lesions on MRI. Multiparametric models could be useful as a supportive decision tool to accurately classify breast lesions, especially for less experienced breast MRI readers.

## Figures and Tables

**Table 1:** BI-RADS descriptors for enhancing lesions.

Mass Lesions	Non-Mass Lesions
Internal enhancement Homogeneous Heterogeneous Rim enhancement Internal septa	Distribution Focal Lineal Regional Segmental Diffuse
Margins Circumscribed Irregular Spiculated	Internal enhancement Homogeneous Heterogeneous Clumped Clustered
Shape Oval Round Irregular	Enhancing kinetics Persistent Plateau Wash-out
Enhancing kinetics Persistent Plateau Wash-out	

**Table 2:** Characteristics of the 93 patients included in the analysis.

Patient Characteristics	Number (Percentage)
Mean age (years; SD)	49 years ± 12 years
Menopausal status	
Pre-menopausal	55 (59.1%)
Post-menopausal	38 (40.9%)
Breast Findings	
Benign	58 (55.8%)
Malignant	46 (44.2%)

**Table 3:** Characteristics of the 104 lesions included in the analysis.

Benign Lesions		Malignant Lesions	
Mass	50 (86.2%)	Mass	35 (76%)
NME	8 (13.8%)	NME	11 (24%)
**Histopathology**		**Histopathology**	
Fibroadenoma or fibro-adenomatoid change	30 (51.8%)	IDC	Histological Grade 1: 4 (8.6%)
Phyllodes tumor	1 (1.7%)		Histological Grade 2: 18 (39·2%)
Adenosis, stromal fibrosis, ductal ectasia, or normal breast parenchyma	10 (17.3%)		Histological Grade 3: 20 (43·6%)
FCC	5 (8.6%)		
ADH or ALH	4 (6.9%)	ILC	2 (4.3%)
PASH	3 (5.2%)		
Papilloma	2 (3.4%)		
Hamartoma	1 (1.7%)		
Fat necrosis	1 (1.7%)	DCIS	2 (4.3%)
Epithelial intraductal proliferation without atypia	1 (1.7%)		

Abbreviations: NME, non-mass enhancement lesion; FCC, fibrocystic changes; ADH, atypical ductal hyperplasia; ALH, atypical lobular hyperplasia; PASH, pseudo-angiomatous stromal hyperplasia; IDC, invasive ductal carcinoma; ILC, invasive lobular carcinoma; DCIS, ductal carcinoma in situ.

**Table 4:** Diagnostic metrics for the performance of radiologists * and radiomics combining different approaches for mass lesions only.

Assessment type	Sensitivity (95% CI)	Specificity (95% CI)	PPV (95% CI)	NPV (95% CI)	Accuracy (95% CI)	AUC (95% CI)
DWI score *	66.7 (50.5–80.4)	83.3 (68.6–93.0)	80.0 (66.3–89.1)	71.4 (61.5–79.7)	75.0 (64.4–83.8)	0.76 (0.65–0.87)
ADC value *	82.9 (66.4–93.4)	67.4 (52.5–80.1)	64.4 (54.1–73.6)	84.6 (72.1–92.1)	73.8 (63.1–82.8)	0.83 (0.75–0.92)
BI-RADS * (Classic DCE-MRI)	100 (90.0–100)	51.0 (36.3–65.6)	59.3 (52.3–66.0)	100 (90.0–100)	71.4 (60.5–80.8)	0.85 (0.78–0.93)
Radiomics DWI data	62.9 (44.9–78.5)	89.8 (77.8–96.6)	81.5 (64.9–91.3)	77.2 (68.5–84.0)	78.6 (68.3–86.8)	0.83 (0.73–0.92)
Radiomics DWI data with DWI score	68.6 (50.7–83.2)	85.7 (72.8–94.1)	77.4 (62.5–87.6)	79.3 (69.8–86.3)	78.6 (68.3–86.8)	0.86 (0.78–0.94)
Radiomics DWI data with ADC value	80.0 (63.1–91.6)	83.7 (70.3–92.7)	77.8 (64.5–87.1)	85.4 (74.9–92.0)	82.1 (72.3–89.7)	0.89 (0.82–0.96)
Radiomics model using individual BI-RADS descriptors for masses	80.0 (63.1–91.6)	91.8 (80.4–97.7)	87.5 (73.0–94.8)	86.5 (76.7–92.6)	86.9 (77.8–93.3)	0.93 (0.88–0.99)
Radiomics DCE data	54.3 (36.7–71.2)	83.7 (70.3–92.7)	70.4 (54.0–82.8)	71.9 (63.6–79.0)	71.4 (60.5–80.8)	0.76 (0.65–0.86)
Radiomics DCE data with BI-RADS	74.3 (56.7–87.5)	79.6 (65.7–89.8)	72.2 (59.1–82.4)	81.3 (70.8–88.6)	77.4 (67.0–85.8)	0.86 (0.78–0.94)
Radiomics DCE data with individual BI-RADS descriptors for masses	80.0 (63.1–91.6)	91.8 (80.4–97.7)	87.5 (73.0–94.8)	86.5 (76.7–92.6)	86.9 (77.8–93.3)	0.95 (0.90–0.99)
Multiparametric MRI (ADC value with BI-RADS) *	82.9 (66.4–93.4)	89.8 (77.8–96.6)	85.3 (71.4–93.1)	88.0 (77.9–93.9)	86.9 (77.8–93.3)	0.93 (0.87–0.99)
Multiparametric radiomics (DWI and DCE data)	65.7 (47.8–80.9)	89.8 (77.8–96.6)	82.1 (66.0–91.6)	78.6 (69.7–85.4)	79.8 (69.6–87.8)	0.89 (0.82–0.96)
Multiparametric radiomics with DWI score and BI-RADS	91.4 (76.9–98.2)	83.7 (70.3–92.7)	80.0 (67.8–88.4)	93.2 (82.1–97.6)	86.9 (77.8–93.3)	0.93 (0.87–0.98)
Multiparametric radiomics with ADC values and individual BI-RADS descriptors for masses	88.6 (73.3–96.8)	93.9 (83.1–98.7)	91.2 (77.4–96.9)	92.0 (82.0–96.7)	91.7 (83.6–96.6)	0.96 (0.92–1.00)

Abbreviations: DWI, diffusion-weighted imaging; DCE, dynamic contrast-enhanced; CI, confidence interval; PPV, positive predictive value; NPV, negative predictive value; AUC, area under the curve; BI-RADS, Breast Imaging Reporting and Database System. * *p* < 0.05.

## Data Availability

The datasets used and analyzed in this study are not publicly available due to patient privacy requirements but are available upon reasonable request from the corresponding author. The code for radiomic feature extraction used in this study is publicly available via the open-source software CERR (https://github.com/cerr/CERR. Accessed on 7 June 2021.).

## Supplementary Materials

The following supporting information can be downloaded at: https://www.mdpi.com/article/10.3390/cancers14071743/s1, Table S1: Summary of imaging protocols and acquisition parameters; Table S2: Summary of DWI protocols and acquisition parameters; Table S3: Summary of radiomics features selected for each model for the analysis of all lesions; Table S4: Summary of radiomics features selected for each model for the analysis of mass only lesions; Table S5: Diagnostic metrics for the performance of radiologists * and radiomics combining different approaches for mass and non-mass lesions; Table S6: Results from radiologist consensus reading regarding DWI suspicion score and BI-RADS descriptors and classification for mass and non-mass lesions; Table S7: Results from radiologist independent reading regarding DWI suspicion score, BI-RADS classification and multiparametric MRI classification for mass and non-mass lesions.

## References

[1] Mann R.M., Kuhl C.K., Moy L. (2019). Contrast-enhanced MRI for breast cancer screening. J. Magn. Reson. Imaging.

[2] Pinker K., Helbich T.H., Morris E.A. (2017). The potential of multiparametric MRI of the breast. Br. J. Radiol..

[3] Mann R.M., Cho N., Moy L. (2019). Breast MRI: State of the art. Radiology.

[4] Mann R.M., Balleyguier C., Baltzer P.A., Bick U., Colin C., Cornford E., Evans A., Fallenberg E., Forrai G., Fuchsjäger M.H. (2015). Breast MRI: EUSOBI recommendations for women’s information. Eur. Radiol..

[5] Zhang Y., Ren H. (2017). Meta-analysis of diagnostic accuracy of magnetic resonance imaging and mammography for breast cancer. J. Cancer Res. Ther..

[6] Veenhuizen S.G.A., de Lange S.V., Bakker M.F., Pijnappel R.M., Mann R.M., Monninkhof E.M., Emaus M.J., de Koekkoek-Doll P.K., Bisschops R.H.C., Lobbes M.B.I. (2021). Supplemental breast MRI for women with extremely dense breasts: Results of the second screening round of the DENSE trial. Radiology.

[7] Gao Y., Reig B., Heacock L., Bennett D.L., Heller S.L., Moy L. (2021). Magnetic resonance imaging in screening of breast cancer. Radiol. Clin. N. Am..

[8] Pinker K., Moy L., Sutton E.J., Mann R.M., Weber M., Thakur S.B., Jochelson M.S., Bago-Horvath Z., Morris E.A., Baltzer P.A. (2018). Diffusion-weighted imaging with apparent diffusion coefficient mapping for breast cancer detection as a stand-alone parameter: Comparison with dynamic contrast-enhanced and multiparametric magnetic resonance imaging. Investig. Radiol..

[9] Daimiel Naranjo I., Lo Gullo R., Saccarelli C., Thakur S.B., Bitencourt A., Morris E.A., Jochelson M.S., Sevilimedu V., Martinez D.F., Pinker-Domenig K. (2020). Diagnostic value of diffusion-weighted imaging with synthetic b-values in breast tumors: Comparison with dynamic contrast-enhanced and multiparametric MRI. Eur. Radiol..

[10] Iima M., Honda M., Sigmund E.E., Ohno Kishimoto A., Kataoka M., Togashi K. (2020). Diffusion MRI of the breast: Current status and future directions. J. Magn. Reson. Imaging.

[11] Partridge S.C., Nissan N., Rahbar H., Kitsch A.E., Sigmund E.E. (2017). Diffusion-weighted breast MRI: Clinical applications and emerging techniques. J. Magn. Reson. Imaging.

[12] Dijkstra H., Dorrius M.D., Wielema M., Pijnappel R.M., Oudkerk M., Sijens P.E. (2016). Quantitative DWI implemented after DCE-MRI yields increased specificity for BI-RADS 3 and 4 breast lesions. J. Magn. Reson. Imaging.

[13] Pinker K., Bogner W., Baltzer P., Gruber S., Bickel H., Brueck B., Trattnig S., Weber M., Dubsky P., Bago-Horvath Z. (2014). Improved diagnostic accuracy with multiparametric magnetic resonance imaging of the breast using dynamic contrast-enhanced magnetic resonance imaging, diffusion-weighted imaging, and 3-dimensional proton magnetic resonance spectroscopic imaging. Investig. Radiol..

[14] Pinker K., Bickel H., Helbich T.H., Gruber S., Dubsky P., Pluschnig U., Rudas M., Bago-Horvath Z., Weber M., Trattnig S. (2013). Combined contrast-enhanced magnetic resonance and diffusion-weighted imaging reading adapted to the “breast imaging reporting and data system” for multiparametric 3-t imaging of breast lesions. Eur. Radiol..

[15] Clauser P., Krug B., Bickel H., Dietzel M., Pinker K., Neuhaus V.-F., Marino M.A., Moschetta M., Troiano N., Helbich T.H. (2021). Diffusion-weighted imaging allows for downgrading MR BI-RADS 4 lesions in contrast-enhanced MRI of the breast to avoid unnecessary biopsy. Clin. Cancer Res. Off. J. Am. Assoc. Cancer Res..

[16] Zhang M., Horvat J.V., Bernard-Davila B., Marino M.A., Leithner D., Ochoa-Albiztegui R.E., Helbich T.H., Morris E.A., Thakur S., Pinker K. (2019). Multiparametric MRI model with dynamic contrast-enhanced and diffusion-weighted imaging enables breast cancer diagnosis with high accuracy. J. Magn. Reson. Imaging.

[17] Baltzer P., Mann R.M., Iima M., Sigmund E.E., Clauser P., Gilbert F.J., Martincich L., Partridge S.C., Patterson A., Pinker K. (2020). Diffusion-weighted imaging of the breast—A consensus and mission statement from the EUSOBI international breast diffusion-weighted imaging working group. Eur. Radiol..

[18] Lee S.H., Shin H.J., Moon W.K. (2021). Diffusion-weighted magnetic resonance imaging of the breast: Standardization of image acquisition and interpretation. Korean J. Radiol..

[19] Leithner D., Bernard-Davila B., Martinez D.F., Horvat J.V., Jochelson M.S., Marino M.A., Avendano D., Ochoa-Albiztegui R.E., Sutton E.J., Morris E.A. (2020). Radiomic signatures derived from diffusion-weighted imaging for the assessment of breast cancer receptor status and molecular subtypes. Mol. Imaging Biol..

[20] Bitencourt A.G., Gibbs P., Rossi Saccarelli C., Daimiel I., Lo Gullo R., Fox M.J., Thakur S., Pinker K., Morris E., Morrow M. (2020). MRI-Based Machine Learning Radiomics Can Predict HER2 Expression Level and Pathologic Response after Neoadjuvant Therapy in HER2 Overexpressing Breast Cancer.

[21] Dong Y., Feng Q., Yang W., Lu Z., Deng C., Zhang L., Lian Z., Liu J., Luo X., Pei S. (2018). Preoperative prediction of sentinel lymph node metastasis in breast cancer based on radiomics of T2-weighted fat-suppression and diffusion-weighted MRI. Eur. Radiol..

[22] Liu Z., Li Z., Qu J., Zhang R., Zhou X., Li L., Sun K., Tang Z., Jiang H., Li H. (2019). Radiomics of multiparametric MRI for pretreatment prediction of pathologic complete response to neoadjuvant chemotherapy in breast cancer: A multicenter study. Clin. Cancer Res. Off. J. Am. Assoc. Cancer Res..

[23] Mayerhoefer M.E., Materka A., Langs G., Häggström I., Szczypiński P., Gibbs P., Cook G. (2020). Introduction to radiomics. J. Nucl. Med. Off. Publ. Soc. Nucl. Med..

[24] Lo Gullo R., Daimiel I., Morris E.A., Pinker K. (2020). Combining molecular and imaging metrics in cancer: Radiogenomics. Insights Imaging.

[25] Tagliafico A.S., Piana M., Schenone D., Lai R., Massone A.M., Houssami N. (2020). Overview of radiomics in breast cancer diagnosis and prognostication. Breast Edinb. Scotl..

[26] Bickelhaupt S., Jaeger P.F., Laun F.B., Lederer W., Daniel H., Kuder T.A., Wuesthof L., Paech D., Bonekamp D., Radbruch A. (2018). Radiomics based on adapted diffusion kurtosis imaging helps to clarify most mammographic findings suspicious for cancer. Radiology.

[27] Bickelhaupt S., Paech D., Kickingereder P., Steudle F., Lederer W., Daniel H., Götz M., Gählert N., Tichy D., Wiesenfarth M. (2017). Prediction of malignancy by a radiomic signature from contrast agent-free diffusion MRI in suspicious breast lesions found on screening mammography. J. Magn. Reson. Imaging.

[28] Ibrahim A., Primakov S., Beuque M., Woodruff H.C., Halilaj I., Wu G., Refaee T., Granzier R., Widaatalla Y., Hustinx R. (2021). Radiomics for precision medicine: Current challenges, future prospects, and the proposal of a new framework. Methods.

[29] Rogers W., Thulasi Seetha S., Refaee T.A.G., Lieverse R.I.Y., Granzier R.W.Y., Ibrahim A., Keek S.A., Sanduleanu S., Primakov S.P., Beuque M.P.L. (2020). Radiomics: From qualitative to quantitative imaging. Br. J. Radiol..

[30] Daimiel Naranjo I., Gibbs P., Reiner J.S., Lo Gullo R., Sooknanan C., Thakur S.B., Jochelson M.S., Sevilimedu V., Morris E.A., Baltzer P.A.T. (2021). Radiomics and machine learning with multiparametric breast MRI for improved diagnostic accuracy in breast cancer diagnosis. Diagnostics.

[31] Apte A.P., Iyer A., Crispin-Ortuzar M., Pandya R., van Dijk L.V., Spezi E., Thor M., Um H., Veeraraghavan H., Oh J.H. (2018). Technical note: Extension of CERR for computational radiomics: A comprehensive MATLAB platform for reproducible radiomics research. Med. Phys..

[32] Johnson W.E., Li C., Rabinovic A. (2007). Adjusting batch effects in microarray expression data using empirical bayes methods. Biostat. Oxf. Engl..

[33] McDonald E.S., Romanoff J., Rahbar H., Kitsch A.E., Harvey S.M., Whisenant J.G., Yankeelov T.E., Moy L., DeMartini W.B., Dogan B.E. (2021). Mean apparent diffusion coefficient is a sufficient conventional diffusion-weighted MRI metric to improve breast MRI diagnostic performance: Results from the ECOG-ACRIN cancer research group A6702 diffusion imaging trial. Radiology.

[34] Rahbar H., Zhang Z., Chenevert T.L., Romanoff J., Kitsch A.E., Hanna L.G., Harvey S.M., Moy L., DeMartini W.B., Dogan B. (2019). Utility of diffusion-weighted imaging to decrease unnecessary biopsies prompted by breast MRI: A trial of the ECOG-ACRIN cancer research group (A6702). Clin. Cancer Res. Off. J. Am. Assoc. Cancer Res..

[35] Avendano D., Marino M.A., Leithner D., Thakur S., Bernard-Davila B., Martinez D.F., Helbich T.H., Morris E.A., Jochelson M.S., Baltzer P.A.T. (2019). Limited role of DWI with apparent diffusion coefficient mapping in breast lesions presenting as non-mass enhancement on dynamic contrast-enhanced MRI. Breast Cancer Res..

[36] Sutton E.J., Huang E.P., Drukker K., Burnside E.S., Li H., Net J.M., Rao A., Whitman G.J., Zuley M., Ganott M. (2017). Breast MRI radiomics: Comparison of computer- and human-extracted imaging phenotypes. Eur. Radiol. Exp..

[37] Truhn D., Schrading S., Haarburger C., Schneider H., Merhof D., Kuhl C. (2018). Radiomic versus convolutional neural networks analysis for classification of contrast-enhancing lesions at multiparametric breast MRI. Radiology.

[38] Lo Gullo R., Daimiel I., Rossi Saccarelli C., Bitencourt A., Gibbs P., Fox M.J., Thakur S.B., Martinez D.F., Jochelson M.S., Morris E.A. (2020). Improved characterization of sub-centimeter enhancing breast masses on MRI with radiomics and machine learning in BRCA mutation carriers. Eur. Radiol..

[39] Mao Y.-J., Lim H.-J., Ni M., Yan W.-H., Wong D.W.-C., Cheung J.C.-W. (2022). Breast tumour classification using ultrasound elastography with machine learning: A systematic scoping review. Cancers.

