# Supplementary material for: Breast Lesion Classification with Multiparametric Breast MRI Using Radiomics and Machine Learning: A Comparison with Radiologists’ Performance
Source: Cancers (Basel). 2022 Mar 29;14(7):1743. doi: 10.3390/cancers14071743 (PMC8997089; doi:10.3390/cancers14071743)
Supplement: Supplementary file 1 [file cancers-14-01743-s001.zip › cancers-1629606-supplementary.pdf]

SUPPLEMENTARY MATERIAL

Contents

Patient Inclusion and Exclusion Criteria 2

Breast MRI Technique 3

Table S1. Summary of imaging protocols and acquisition parameters. 3

Table S2. Summary of DWI protocols and acquisition parameters. 4

Top five radiomics parameters selected to develop each model for the separation of benign and malignant lesions 5

Table S3. Summary of radiomics features selected for each model for the analysis of all lesions. 5

Table S4. Summary of radiomics features selected for each model for the analysis of mass only lesions. 5

Radiologist Performance vs Radiomics Coupled with ML for the Classification of All Lesions (Mass and Non-Mass Enhancement) 7

Table S5. Diagnostic metrics for the performance of radiologists* and radiomics combining different approaches for mass and non-mass lesions. 8

Table S6. Results from radiologist consensus reading regarding DWI suspicion score and BI-RADS descriptors and classification for mass and non-mass lesions. 9

Table S7. Results from radiologist independent reading regarding DWI suspicion score, BI-RADS classification and multiparametric MRI classification for mass and non-mass lesions. 10

Patient Inclusion and Exclusion Criteria

The inclusion criteria were as follows: women aged 18 years and above with enhancing lesions on breast MRI that were classified as suspicious on routine clinical reads (Breast Imaging-Reporting and Data System (BI-RADS) 4/5), and who subsequently underwent image-guided biopsy according to BI-RADS criteria. If no correlate for the suspicious enhancing abnormality could be identified on second-look ultrasound, the lesion was biopsied under MRI guidance. If there was a correlate on ultrasound, the lesion was biopsied under ultrasound guidance. Patients with breast implants and those whose MRI examinations did not contain good quality images or DWI sequences were excluded.

Breast MRI Technique

In this study, two different types of MRI scanners were used. The examinations at Center 1 were performed on 3 T MRI scanners (GE Discovery 750, GE Healthcare) using 8-channel (13/93 examinations, 14%) or 16-channel breast coils (Sentinelle coils) (20/93 examinations, 21%) and included fat-suppressed T2-weighted fast spin echo imaging and fat-suppressed 3D T1-weighted imaging using differential subsampling with Cartesian ordering (DISCO) both before and after contrast agent injection (0.1 mmol gadobutrol/kg body weight). DW images were acquired using two encoding schemes: single-shot echo-planar with parallel imaging array spatial sensitivity encoding technique (ASSET) (22/33 examinations, 66.7%), and multishot multiplexed sensitivity-encoding (MUSE) (11/33 examinations, 33.3%). The examinations at Center 2 were acquired on a 3 T MRI scanner (Tim Trio, Siemens) using 4-channel breast coils (InVivo) (60/93 examinations, 65%) and included fat-suppressed T2-weighted turbo spin echo imaging, fat-suppressed DCE T1-weighted imaging before and after contrast injection (0.1 mmol gadoterate meglumine/kg body weight), and readout-segmented echo planar imaging DWI.

In all examinations, DW images were acquired before injection of the contrast agent. Apparent diffusion coefficient (ADC) mapping was generated using built-in software. The MRI acquisition parameters for both types of scanners are summarised in **Tables S1 and S2.**

Table S1. Summary of imaging protocols and acquisition parameters.

Scanner	3 T MRI Tim Trio, Siemens			3 T GE Discovery 750, GE		
Sequence	T2-weighted turbo spin echo	T1-weighted VIBE	T1-weighted turbo FLASH-3D	T2-weighted fast spin echo	T1-weighted	T1-weighted DISCO
Fat suppression	Nonselective inversion	Frequency selective	Frequency selective	Inversion recovery	Inversion recovery	Inversion recovery
Repetition time (msec)	4800	3·61	877	6460	7·9	7·9
Echo time (msec)	61	1·4	3·82	104·1	4.3	4.3
Matrix size (mm)	512 × 512	512 × 512	512 × 512	512 × 512	512 × 512	512 × 512
Resolution (mm)	1 × 1 × 4	1.7 × 1.7 × 1.7	1 × 1 × 1	1 × 1 × 3	1 × 1 × 1	1 × 1 × 1
Parallel imaging	GRAPPA 2	GRAPPA 2	GRAPPA 2	ASSET	ASSET	ASSET
Image acquisition time (min:sec)	2:26	0:13	2:00	2:32	1:30	4:30
Number of lesions	65			39		

Abbreviations: ASSET, array spatial sensitivity encoding technique; VIBE, Volumetric interpolated breath-hold examination; FLASH, fast low-angle shot; DISCO, Differential Sub-sampling with Cartesian Ordering

Table S2. Summary of DWI protocols and acquisition parameters.

Scanner	3 T Tim Trio, Siemens	3 T GE Discovery 750, GE	
Sequence	Axial readout segmented echo-planar imaging	Axial single-shot DWI ASSET echo planar imaging	Axial multiplexed sensitivity-encoding DWI
Diffusion directions	Three-direction trace	Three-direction trace	Three-direction trace
b value (s/mm2)	0, 850	0, 800	0, 800
Fat suppression	Inversion recovery; gradient reversal	Inversion recovery	Inversion recovery
Repetition time (msec)	8000	6000	2000-17000
Echo time (msec)	Minimum	Minimum	Minimum
Inversion time (msec)	210	210	210
Field of view (mm)	360 × 202	340 × 320	340 × 320
Matrix	172 × 96	256 × 256	300 × 300
Section thickness (mm)	5	3.9	3.9
Intersection gap (mm)	5	3.9	3.9
Nº of readout segments	5	1	4
Nº of sections	24	34	34
Phase-encoding direction	anteroposterior	anteroposterior	anteroposterior
Time of scan (min:s)	2:56	4:02	6:04
Number of lesions	65	21	18

Abbreviations: ASSET, array spatial sensitivity encoding technique; DWI, diffusion-weighted imaging

Top five radiomics parameters selected to develop each model for the separation of benign and malignant lesions

Table S3. Summary of radiomics features selected for each model for the analysis of all lesions.

	Feature 1	Feature 2	Feature 3	Feature 4	Feature 5
DWI Radiomics	glnNorm (SZM)	minimum (FO)	run emphasis (RLM)	rms (FO)	correlation (GLCM)
DWI Radiomics with DWI score	DWI score	glnNorm (SZM)	minimum (FO)	run emphasis (RLM)	rms (FO)
DWI Radiomics with ADC value	ADC value	glnNorm (SZM)	correlation (GLCM)	run emphasis (RLM)	joint entropy (GLCM)
DCE Radiomics	entropy (FO)	strength (NGTDM)	coarseness (NGTDM)	rlnNorm (RLM)	joint maximum (GLCM)
DCE Radiomics with BI-RADS	BI-RADS score	entropy (FO)	inverse variance (GLCM)	zln (SZM)	strength (NGTDM)
Multiparametric Radiomics	entropy (FO DCE)	strength (NGTDM DCE)	coarseness (NGTDM DCE)	minimum (FO ADC)	glnNorm (SZM ADC)
Multiparametric Radiomics with DWI score and BIRADS	BI-RADS	entropy (FO DCE)	DWI score	glnNorm (SZM ADC)	minimum (FO ADC)
Multiparametric Radiomics with ADC value and BIRADS	BI-RADS	ADC value	entropy (FO DCE)	inverse variance (GLCM DCE)	strength (NGTDM DCE)

Abbreviations: FO, first order; GLCM, gray level cooccurrence matrix; SZM, size zone matrix; NGTDM, neighborhood gray tone difference matrix; glnNorm, gray level nonuniformity.

Table S4. Summary of radiomics features selected for each model for the analysis of mass only lesions.

	Feature 1	Feature 2	Feature 3	Feature 4	Feature 5
DWI Radiomics	minimum (FO)	glnNorm (SZM)	correlation (GLCM)	rms (FO)	90th percentile (FO)
DWI Radiomics with DWI score	minimum (FO)	glnNorm (SZM)	correlation (GLCM)	DWI score	rms (FO)

DWI Radiomics with ADC value	ADC value	correlation (GLCM)	glnNorm (SZM)	minimum (FO)	lglze (SZM)
DCE Radiomics	entropy (FO)	joint maximum (GLCM)	strength (NGTDM)	sre (RLM)	rlnNorm (RLM)
DCE Radiomics with BI-RADS	BIRADS	entropy (FO)	joint maximum (GLCM)	strength (NGTDM)	coarseness (NGTDM)
DCE Radiomics with BI-RADS individual descriptors	Margin	Kinetics	entropy (FO)	strength (NGTDM)	coarseness (NGTDM)
Multiparametric Radiomics	glnNorm (SZM ADC)	entropy (FO DCE)	minimum (FO ADC)	rms (FO ADC)	correlation (GLCM ADC)
Multiparametric Radiomics with DWI score and BIRADS	BIRADS	entropy (FO DCE)	glnNorm (SZM ADC)	minimum (FO ADC)	correlation (GLCM ADC)
Multiparametric Radiomics with ADC value and BI-RADS descriptors	Margin	Kinetics	entropy (FO DCE)	ADC value	correlation (GLCM ADC)

Abbreviations: FO, first order; GLCM, gray level cooccurrence matrix; SZM, size zone matrix; rms, root mean square; glnNorm, gray level non-uniformity normalized.

Radiologist Performance vs Radiomics Coupled with ML for the Classification of All Lesions (Mass and Non-Mass Enhancement)

The performance of radiologist consensus reading, as well as that of different models for the classification of both all lesions, are shown in **Tables S5**. **Table S6** shows the results of radiologist consensus reading regarding BI-RADS assessment category, BI-RADS descriptors, and DWI suspicion score, stratified by benign and malignant lesions. **Table S7** shows the results of radiologist independent reading regarding BI-RADS assessment category, BI-RADS descriptors, and DWI suspicion score, stratified by benign and malignant lesions.

For the classification of lesions based on DWI, the radiomics DWI data model that utilised DWI-derived features alone had a diagnostic accuracy (73.1%, CI: 63.5%–81.3%) that was lower, albeit not significantly, compared with that of the DWI score (77.9%, CI: 68.7%–85.4%) or the ADC value (76.0%, CI: 66.6%–83.8%) as assessed by radiologists ($p > 0.35$ for both). When the model combined DWI-derived features and ADC value, i.e., radiomics DWI data with ADC value model, the diagnostic accuracy improved to 79.8% (CI: 70.8%–87.0%), albeit this was not significantly different from the diagnostic accuracy of the radiomics DWI data model ($p = 0.09$), the DWI score ($p = 0.70$), or the ADC value ($p = 0.35$).

For the classification of lesions based on DCE-MRI, the radiomics DCE data with BI-RADS model yielded the highest diagnostic accuracy (83.7%, CI: 75.1%–90.2%), although it was not significantly different than the diagnostic accuracy of BI-RADS (classic DCE-MRI) scoring as assessed by radiologists (74.0%, CI: 64.5%–82.1%) ($p = 0.05$) or the radiomics DCE data model that utilised DCE-derived features alone (76.0%, CI: 66.6%–83.8%) ($p = 0.11$).

For the classification of lesions based on multiparametric assessment of DWI and DCE data, there were no significant differences in diagnostic accuracy between the multiparametric radiomics (DWI and DCE data) model (77.9%, CI: 68.7%–85.4%) and the multiparametric MRI (ADC value with BI-RADS) assessment by radiologists (85.6%, CI: 77.3%–91.7%) ($p > 0.15$). Multiparametric radiomics with DWI score and BI-RADS (88.5%, CI: 80.7%–93.9%) and multiparametric radiomics with ADC values and BI-RADS (88.5%, CI: 80.7%–93.9%) models showed significant improvements in diagnostic accuracy when compared with the multiparametric radiomics (DWI and DCE data) model ($p = 0.01$ and $p = 0.02$, respectively). However, no significant differences were noted between the multiparametric radiomics model with ADC values and BI-RADS, and radiologist MRI (ADC value with BI-RADS) multiparametric assessment by radiologists ($p = 0.39$).

Table S5. Diagnostic metrics for the performance of radiologists* and radiomics combining different approaches for mass and non-mass lesions.

	Sensitivity (95% CI)	Specificity (95% CI)	PPV (95% CI)	NPV (95% CI)	Accuracy (95% CI)	AUC (95% CI)
DWI score*	84.8 (71.1–93.7)	72.4 (59.1–83.3)	70.9 (61.2–79.0)	85.7 (74.9–92.4)	77.9 (68.7–85.4)	0.80 (0.71–0.89)
ADC value*	84.8 (71.1–93.7)	69.0 (55.5–80.5)	68.4 (59.2–76.4)	85.1 (73.9–92.0)	76.0 (66.6–83.8)	0.84 (0.76–0.92)
BI-RADS* (classic DCE-MRI)	100 (92.3–100)	53.5 (39.9–66.7)	63.0 (56.4–69.2)	100 (92.3 –100)	74.0 (64.5–82.1)	0.86 (0.80–0.93)
Radiomics DWI data	67.4 (52.0–80.5)	77.6 (64.7–87.5)	70.5 (58.7–80.0)	75.0 (65.9–82.3)	73.1 (63.5–81.3)	0.80 (0.72–0.89)
Radiomics DWI data with DWI score	76.1 (61.2–87.4)	79.3 (66.7–88.8)	74.5 (63.2–83.2)	80.7 (71.1–87.7)	77.9 (68.7–85.4)	0.85 (0.77–0.93)
Radiomics DWI data with ADC value	80.4 (66.1–90.6)	79.3 (66.7–88.8)	75.5 (64.6–83.9)	83.6 (73.7–90.3)	79.8 (70.8–87.0)	0.84 (0.76–0.92)
Radiomics DCE data	76.1 (61.2–87.4)	75.9 (62.8–86.1)	71.4 (60.6–80.2)	80.0 (70.1–87.2)	76.0 (66.6–83.8)	0.83 (0.75–0.91)
Radiomics DCE data with BI-RADS	82.6 (68.6–92.2)	84.5 (72.6–92.7)	80.9 (69.5–88.7)	86.0 (76.4–92.1)	83.7 (75.1–90.2)	0.92 (0.87–0.98)
Multiparametric MRI (ADC value with BI-RADS) *	89.1 (76.4–96.4)	82.8 (70.6–91.4)	80.4 (69.8–87.9)	90.6 (80.6–95.7)	85.6 (77.3–91.7)	0.93 (0.88–0.97)
Multiparametric radiomics (DWI and DCE data)	82.6 (68.6–92.2)	74.1 (61.0–84.7)	71.7 (61.6–80.0)	84.3 (73.8–91.1)	77.9 (68.7–85.4)	0.80 (0.71–0.89)
Multiparametric radiomics with DWI score and BI-RADS	89.1 (76.4–96.4)	87.9 (76.7–95.0)	85.4 (74.4–92.2)	91.1 (81.6–95.9)	88.5 (80.7–93.9)	0.93 (0.89–0.98)
Multiparametric radiomics with ADC values and BI-RADS	95.7 (85.2–99.5)	82.8 (70.6–91.4)	81.5 (71.4–88.6)	96.0 (86.0–98.9)	88.5 (80.7–93.9)	0.96 (0.93–0.99)

Abbreviations: DWI, diffusion-weighted imaging; DCE, dynamic contrast-enhanced; CI, confidence interval; PPV, positive predictive value; NPV, negative predictive value; AUC, area under the curve; BI-RADS, Breast Imaging Reporting and Database System

Table S6. Results from radiologist consensus reading regarding DWI suspicion score and BI-RADS descriptors and classification for mass and non-mass lesions.

	**Consensus**	
**Lesion type**	Malignant (n = 46)	Benign (n = 58)
**BI-RADS**		
2	0	12 (20.7)
3	0	19 (32.7)
4	27 (53.7)	27 (46.6)
5	19 (46.3)	0
**BI-RADS descriptors for mass lesions (85)**		
Internal enhancement		
Homogeneous	5 (14.2)	16 (32)
Heterogeneous	22 (62.8)	24 (48)
Rim enhancement	8 (23)	2 (4)
Internal dark septa	0	8 (16)
Margins		
Circumscribed	0	22 (44)
Irregular	19 (54.3)	28 (56)
Spiculated	16 (45.7)	0
Shape		
Oval	7 (30)	15 (30)
Round	7 (30)	14 (28)
Irregular	21 (60)	21 (42)
Enhancing Kinetics		
Persistent	8 (22.8)	28 (56)
Plateau	14 (40)	17 (34)
Wash-out	13 (37.2)	5 (10)
**BI-RADS descriptors for non-mass lesions (19)**		
Distribution		
**Focal**	1 (9.1)	6 (75)
**Lineal**	1 (9.1)	1 (12.5)
**Regional**	3 (27.3)	0
**Segemental**	5 (45.4)	1 (12.5)
**Diffuse**	1 (9.1)	0
Internal Enhancement		
**Homogeneous**	4 (36.4)	3 (37.5)
**Heterogeneous**	5 (45.4)	3 (37.5)
**Clumpled**	0	0
**Clustered**	2 (18.2)	2 (25)
**Enhancing Kinetics**		
**Persistent**	2 (18.2)	5 (62.5)
**Plateau**	4 (36.4)	2 (25)
**Wash-out**	5 (45.4)	1 (12.5)
**DWI suspicion score**		
**1**	1 (2.2)	8 (13.8)

**2**	4 (8.8)	5 (8.6)
**3**	2 (4.4)	29 (50)
**4**	20 (43.4)	13 (22.5)
**5**	19 (41.2)	3 (5.1)

Abbreviations: DWI, diffusion-weighted imaging, Breast Imaging Reporting and Database System
*Figure in brackets are percentages unless otherwise specified.

Table S7. Results from radiologist independent reading regarding DWI suspicion score, BI-RADS classification and multiparametric MRI classification for mass and non-mass lesions.

	**Radiologist 1**		**Radiologist 2**	
**Lesion type**	Malignant 46	Benign 58	Malignant	Benign
**BI-RADS**				
2	0	16 (27.5)	0	9 (15.5)
3	1 (2.2)	11 (19)	0	21 (36.2)
4	26 (56.5)	31 (53.5)	26 (56.5)	28 (48.3)
5	19 (41.3)	0	20 (43.5)	0
**DWI suspicion score**				
1	1 (2.2)	11 (18.9)	1 (2.2)	2 (3.4)
2	4 (8.7)	4 (7)	6 (13)	9 (15.5)
3	4 (8.7)	29 (50)	3 (6.5)	25 (43.3)
4	24 (52.2)	12 (20.7)	17 (36.9)	11 (18.9)
5	13 (28.2)	2 (3.4)	19 (41.4)	11 (18.9)
**Multiparametric MRI classification**				
Bening	6 (13)	46 (79.3)	6 (13)	41 (70.7)
Malignant	40 (87)	12 (20.7)	40 (87)	17 (29.3)

Abbreviations: DWI, diffusion-weighted imaging; BI-RADS, Breast Imaging Reporting and Database System
*Figure in brackets are percentages unless otherwise specified.
